# Plot Locator: An app for locating plots in the field

**DOI:** 10.1002/aps3.11311

**Published:** 2019-12-20

**Authors:** Jere A. Boudell, Beth A. Middleton

**Affiliations:** ^1^ Department of Biology Clayton State University 2000 Clayton State Boulevard Morrow Georgia 30260 USA; ^2^ U.S. Geological Survey, Wetland and Aquatic Research Center 700 Cajundome Boulevard Lafayette Louisiana 70506 USA

**Keywords:** distance measurement, field techniques, mobile app, outdoor app

## Abstract

**Premise:**

One of the challenges in field biology is locating previously sampled plots. The Plot Locator app was developed to assist field biologists with plot identification and location, with or without GPS or online connectivity.

**Methods and Results:**

The Plot Locator Android app helps users locate field plots by creating a searchable database that stores study area information, such as site/plot names and numbers, distances from landmarks, optional cardinal directions and GPS coordinates, and field notes. A GPS assist and Google Maps can also be used with the app when connectivity is available. All study location data and field notes are stored in a downloadable CSV file on the user's device.

**Conclusions:**

The Plot Locator app provides a comprehensive searchable database of study area information, plot location information, and location aids, which are easily accessed in the field.

Locating previously sampled field sites and plots can be a daunting task for field biologists. Site markers such as flags, buried metal markers, and/or GPS satellites are commonly used to mark study sites, but cannot always be used; for example, the use of permanent location aids may be prohibited in studies conducted on managed sites. Researchers working in remote locations or without access to highly accurate GPS receivers may not be able to make use of GPS, and even if a biologist could use a GPS device, its accuracy may be impacted by interference from trees and other blockages (GPS.gov, 2017). Re‐locating smaller plots (e.g., 1 m^2^) can be incredibly difficult, particularly in densely vegetated sites. In these conditions, field biologists typically use extensive field notes stored in notebooks and measurement tapes to measure distance from landmarks to plots. Additionally, field biologists often sample plots out of order based on field conditions; for example, a researcher may choose to sample sites in different study areas that border each other (e.g., one river channel site, a riverbank site, and a floodplain site) vs. sampling all sites for a single study habitat (e.g., all river channel sites, then all bank sites). This approach can result in disjointed plot location notes. Together, the various field conditions, differences in plot location methods (e.g., tapes, location aids, GPS), and disjointed notes can make it challenging to find study plots again in the future.

Mobile devices are powerful computers with many useful sensors, which can function with or without a connection to a cell tower, satellite, or Wi‐Fi signal. Mobile apps can use this technology to assist biologists in the field with a variety of tasks such as organism identification (iNaturalist [https://www.inaturalist.org/], Map of Life [https://www.mol.org/mobile#/]), data collection and storage (iNaturalist, Open Data Kit [https://opendatakit.org/]), and distance measurements (GPS Status [https://mobiwia.com/gpsstatus/#what-does-it-do], Planimeter [http://www.vistechprojects.com/app/planimeter]) (Palumbo, 2012; Boudell, 2013; Teacher et al., 2013; Maya‐Lastra, 2016). Based on our years of field experience and our expertise in ecological app development, we developed Plot Locator to assist field biologists with plot identification and re‐location. Plot Locator is an app for Android mobile devices that does not require GPS or online connectivity. Other mapping and tracking apps such as Fieldmap (https://www.fieldmap.cz/) or OruxMaps (https://www.oruxmaps.com/cs/en/) require location services such as a Wi‐Fi signal, cell towers, and/or GPS satellites to track locations, and therefore may not be helpful when biologists lose connectivity. Plot Locator records site information, as well as the distance and number of steps between plots and user‐selected landmarks. The app also records field notes and saves all location data in a database file on the user's mobile device. When users want to locate their plots again, the app can be used to search the user's database file and display the plot location information. An optional digital compass provides cardinal directions to enrich notes and assist with plot location. When a cell tower, satellite, or Wi‐Fi signal is available, users can activate an optional GPS assist and use Google Maps to help identify a plot location; however, if biologists always have access to location services at their field sites, mapping apps, such as those mentioned above, may provide a better mapping user experience.

The Plot Locator app can help biologists locate field plots without relying on external location services such as GPS, enabling the calculation of distances and ultimately providing an easily searchable alternative tool to help users locate field sites and plots.

## METHODS AND RESULTS

### Design

**Figure 1:**
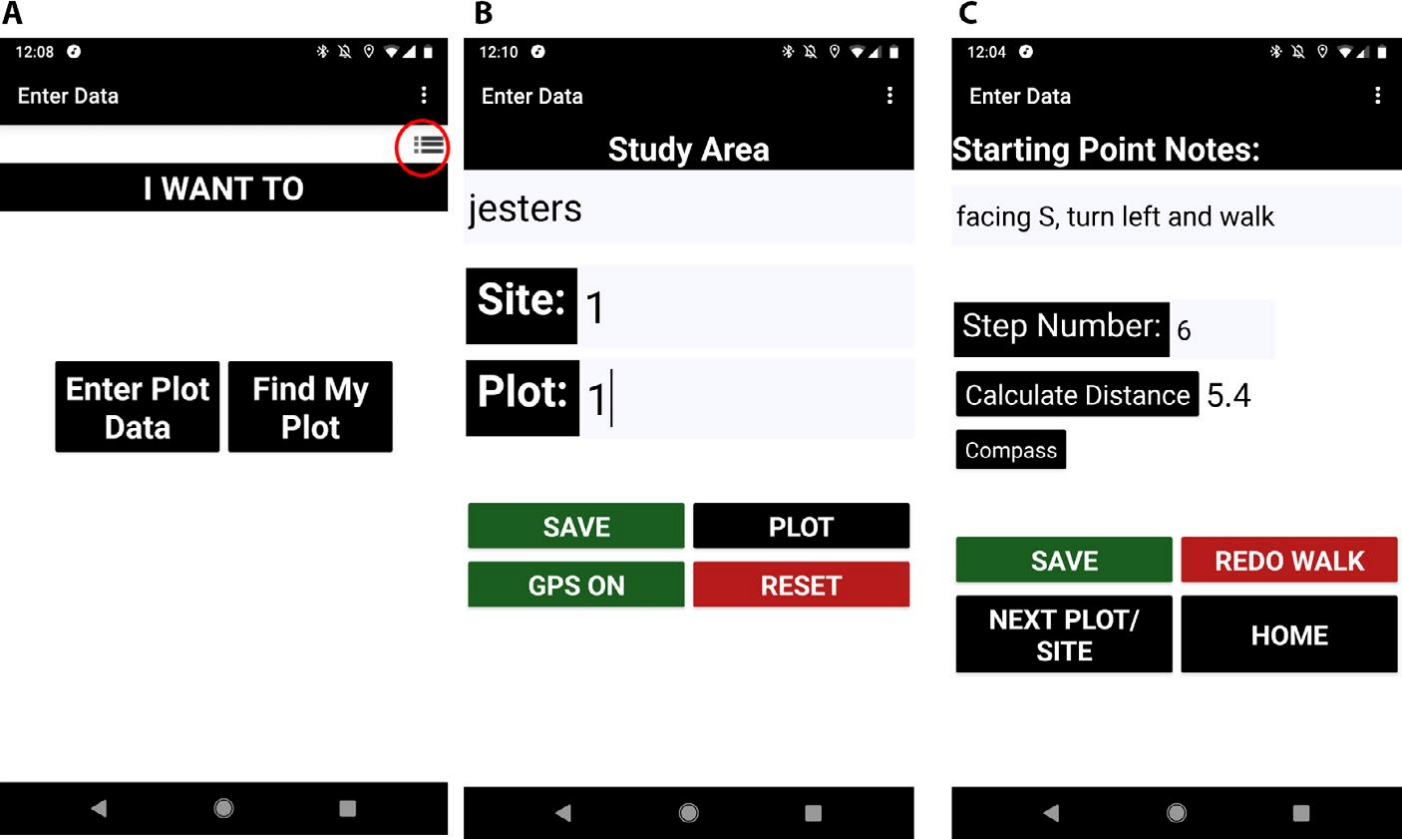
Screenshots of example screens in the Plot Locator app. (A) Plot Locator home screen. The Options icon is circled in red. The options include Export, Delete, and Create New File, as well as a link to the Plot Locator User Guide. (B) Plot Locator “Enter Data” screen. General site information is entered on this screen. An optional GPS assist is also available. (C) Plot Locator “Enter Data” or “Notebook” screen. The user enters starting point notes and step numbers. The app calculates the distance once the “Calculate Distance” button is pressed. Distance is determined using the step number and the user's stride length. An optional compass is available when the user clicks “Compass.”

The Plot Locator app was developed with MIT App Inventor 2 (Pokress and Dominguez Veiga, 2013; Wolber et al., 2014) and Thunkable (https://thunkable.com/), using design principles that maximize usability for biologists working in field conditions. Contrasting colors, principally black and white, and large text and buttons were used to increase visibility in outdoor conditions (Fig. 1A–C) (Google, 2016). Many processes were incorporated into the app to protect the integrity of the user's field data. Actions such as clicking “Save” in the data collection process will not function unless the user has entered data, preventing them from inadvertently saving zero data (e.g., saving a blank entry box). Warning notifications are triggered, for example, when users are about to leave a data collection screen for the home screen. Button colors were selected to encourage users to save data (green) or to be cautious (red), such as when resetting site/plot names and numbers (Fig. 1B, C). Notifications alert the user when data are saved. Another protective measure implemented in the app was to position the buttons away from the bottom of the screen where thumbs typically rest. This measure also allows the app to function when users change their devices from a portrait to landscape orientation, which may be a preferred orientation on smaller mobile devices. Additionally, the app was created using responsive design techniques, which allow the app to resize to varying screen sizes. Together, these design approaches increase usability in outdoor conditions and help prevent data loss, which could easily happen in the field when biologists are tired and accidentally click the wrong button.

### Step counter, notes, and compass

The Plot Locator app makes use of mobile technology to help field biologists with or without a connection to a cell phone tower or GPS satellites. To estimate location without GPS, the Plot Locator app employs a “notebook” for field notes and a manual step counter to determine the distance between the plot and a landmark (Fig. 1C). Users enter their stride length (in meters) to estimate distance (in meters), which is calculated using the user's step number (stride length × step number). If users would like to enter a new stride length because another person is going to use the app on the same device during the same field session, they can simply close and reopen the app and then select the same file. If users would like to use step number to function as distance (e.g., they wish five steps to equal 5 m in distance), then users can enter “1” for their stride length. Once stride length is saved, the “Step Counter” screen appears (Fig. 1C).

Users enter the starting point or landmark in their field notes, which is used to help estimate plot location. The app also makes use of the magnetometer sensor in most mobile devices, enabling the use of an optional digital compass to determine cardinal directions in relation to magnetic north (Fig. 1C) (Pura Vida Apps, 2018). To use the digital compass, users click the “Compass” button, which opens the “Compass” screen. For example, a field biologist might use a large old oak tree as their starting point, and could enter “Face W with back to oak tree” into the “Starting Point Notes” textbox. The user would then begin walking from their starting position until they reach their study plot, where they would enter the number of steps taken to reach the plot and click the “Calculate Distance” button. The calculated distance and number of steps will appear on the screen. If the user is unhappy with their step count, they can click “Redo Walk” to retake the reading. Once the user is satisfied with their step and distance information, they should click “Save” to record the starting point notes, step number, and distance. Clicking the “Back” button returns the user to the prior screen. Notifications alert the user once their data have been saved to the database.

**Figure 2:**
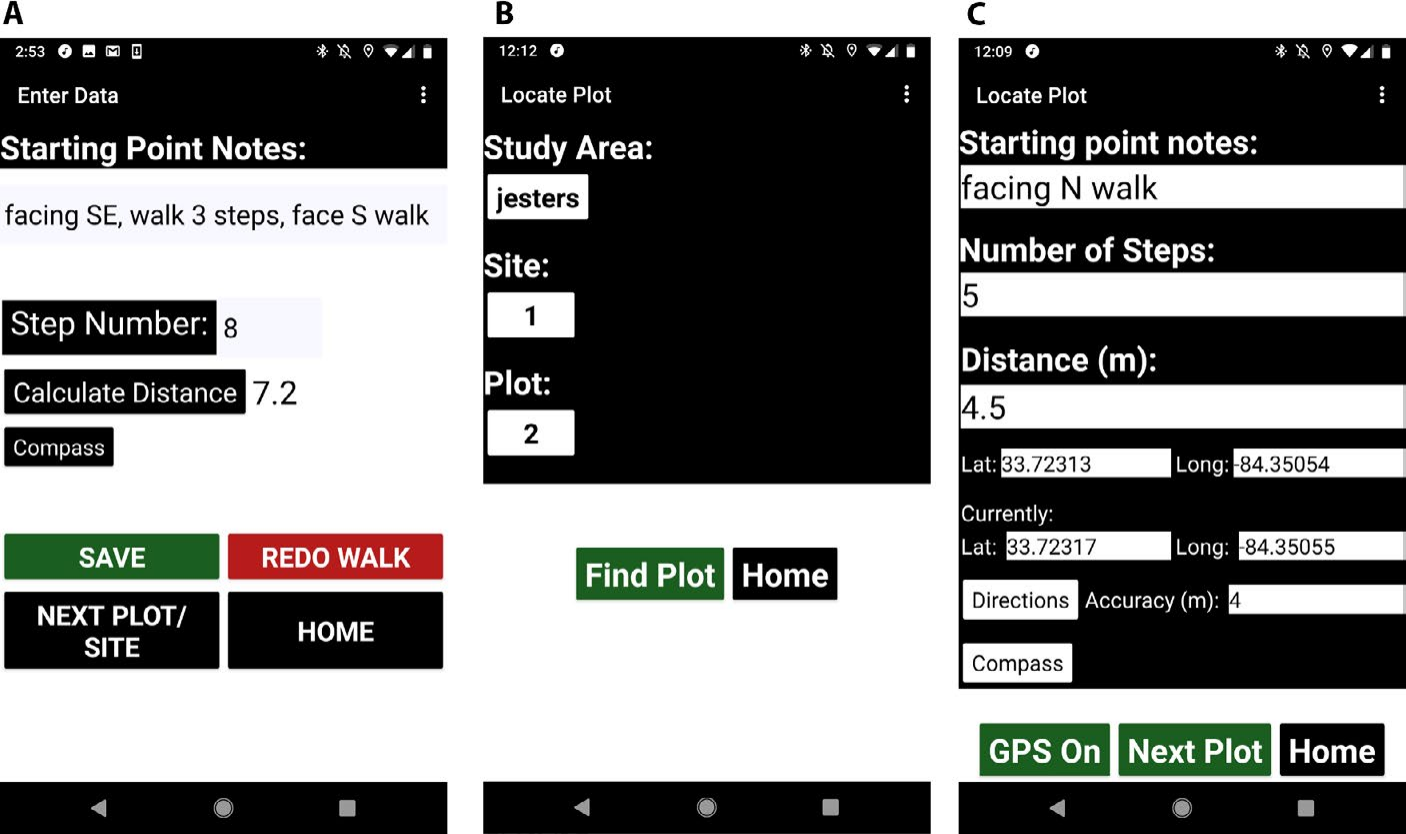
Screenshots showing setting and accessing plot location data in the Plot Locator app. (A) Example of the entry of multiple landmarks or starting points for a single plot. The user entered their first set of starting point notes and step number, and then clicked “Calculate Distance.” In this example, the user entered “3” steps rather than the number of steps and distance. To enter the second starting point (here “face S walk”), the user simply edited their notes. To obtain the second step number and distance, the user clicked “Redo Walk” and selected the option to “Keep Notes.” The user then entered “8” for step number and pressed “Calculate Distance.” To complete the process, the user clicked “Save.” To view the data row for this example, see Table 1, RNP row 3. (B) Plot Locator “Locate Plot” drop‐down menus for study area, site, and plots. (C) Plot Locator “Locate Plot” data screen with GPS assist on. The latitude and longitude of the plot location and the current position are displayed, alongside the current GPS accuracy. Google Maps is accessible via the “Directions” button. The compass is also available from this screen.

Biologists who want to enter multiple starting points or landmarks for a single plot will need to modify the data entry process. The user will need to enter their starting point or landmark description into the “Starting Point Notes” textbox, enter their step number into the “Step Number” textbox, click the “Calculate Distance” button, and then enter the calculated distance and step number into the “Starting Point Notes” textbox next to their starting point description. Instead of clicking “Save,” the user will need to click “Redo Walk” and select the option to “Keep Notes.” They will then be able to add their new starting point or landmark to the “Starting Point Notes” textbox, determine the step number and distance from the new starting point, and add the new step number and distance to their Starting Point Notes. Users can repeat this process until they have completed their readings for the plot; for example, a user with a 0.9‐m stride length could enter in the “Starting Point Notes” textbox, “face S at red oak, walk 5 steps/4.5 m, face N, walk 3 steps/2.7 m” or “facing SE, walk 3 steps, face S, walk” and enter the final step number and calculate the distance (Fig. 2A) (see Table 1, RNP row 3). Once the user has completed their entry for the plot, they should click “Save.”

**Table 1:** Sample of a CSV table created using the Plot Locator app.

Study area	Date	Site	Latitude	Longitude	Plot	Stride length (m)	Start point	Distance (m)	Steps
RNP	Nov 29 2018	1	32.35934	–84.20498	1	0.9	Facing N, walk	4.5	5
RNP	Nov 29 2018	1	33.35928	–84.20559	2	0.9	Facing S, turn left and walk	5.4	6
RNP	Nov 29 2018	2	33.35819	–84.20531	1	0.9	Facing SE, walk 3 steps, face S, walk	7.2	8
RNP	Nov 29 2018	2	33.35793	–84.20491	2	0.9	Facing E, walk	8.1	9
Jesters	Nov 29 2018	1	33.36033	–84.20546	1	0.9	Facing E, walk	6.3	7
Jesters	Nov 29 2018	1	33.3601	–84.20532	2	0.9	Facing N, walk	11.7	13
Jesters	Nov 29 2018	2	33.33076	–84.20561	1	0.9	Facing W, walk	3.6	4
Jesters	Nov 29 2018	2	33.33127	–84.20569	2	0.9	Facing NE, walk 5 steps, then face W, walk	7.2	8

Note

Jesters = Jesters Creek; RNP = Reynolds Nature Preserve.

### Data files

All data are saved in a downloadable CSV file on the SD card (or emulated SD card) of the user's mobile device (Table 1). Saving the file to the user's device allows the real‐time creation of a database of plot location information without the need for online connectivity or a database service. Users can add to previously created database files using the “Get File” function on the “File Name” screen. Adding to a previously created database for a project avoids cluttering the mobile device with multiple files and keeps data organized for easier access when biologists want to use the app to re‐locate plots in the field. Users can also export or delete Plot Locator files by accessing the appropriate functions, which are available via the Options icon at the top of the app on the home and “File” screens (Fig. 1A).

### Finding plots

To use Plot Locator to locate field plots, users select “Find My Plot” from the home screen (Fig. 1A). After the user has selected the name of the file stored on the SD card of their mobile device, the “Locate Plot” screen appears. Users select the name of the study area, site name or number, and plot number from the drop‐down boxes (Fig. 2B). Only the sites and plots associated with the selected study area will appear. The study area, site name or number, and plot number lists are also searchable via the search bar. Once the desired information has been selected, the user clicks “Find Plot.” The Plot Locator app will then search the user's database file for the starting point notes, number of steps, and distance data. If the data are not found, a notice appears to remind users to select the study area first and to check they have used the correct search terms for the study area, site name or number, and plot selections. All location data fitting the search criteria will be displayed on the screen (Fig. 2C). The user can then use this information to locate their study plots. The digital compass is also available from this screen. If a GPS signal is available, the user can use the GPS assist by clicking the “GPS Off” button to turn the GPS on. The GPS coordinates of the plot, if previously recorded, and the current GPS accuracy will appear on the screen along with the user's current location. To locate the next plot, users simply click “Next Plot.” See the “GPS assist” section below and the Plot Locator User Guide for more details about how the GPS assist works.

### GPS assist

For users with GPS access, the Plot Locator app can utilize a GPS sensor as well as other location services modes, as determined by the user's mobile device settings. Users with a GPS receiver in their mobile device have the option of using a GPS assist and Google Maps when GPS is available (Figs. 1B, 2C). Currently, for most mobile devices, GPS is typically accurate to within 4.9 m (van Diggelen and Enge, 2015; van Diggelen et al., 2018); however, the new 5G mobile networks will increase this accuracy, depending on the user's device and access to the network (del Peral‐Rosado et al., 2018).

The GPS accuracy of an individual's mobile device varies due to the type of GPS receiver, the use of the location services mode, and environmental obstructions (GPS.gov, 2017; Google Developers, 2018; van Diggelen et al., 2018). To provide the best accuracy while reducing battery drain, Android uses a combination of GPS satellites, cell towers, wireless network triangulation, and a variety of sensors to determine location (Yang et al., 2010; Google Developers, 2018). This combination of location strategies, as of Android 9.0, is in use when Location Accuracy is turned on. If users want to ensure that their device is only using GPS, they will need to modify their location accuracy settings by going to Settings > Security & Location > Advanced > Google Location Accuracy and turning off Location Accuracy. Users with older versions of Android installed will need to determine how to modify their location services based on their specific version of Android (see the Plot Locator User Guide [available with the app and source code at https://github.com/jboudell/PlotLocator]). The option of having users select how their device determines location allows for flexibility in the field in terms of the availability of location services (e.g., GPS, cell towers) and battery use. Using location services increases battery drain (Google Developers, 2018). Users who do not wish to use GPS assist can simply turn off GPS in the app and/or turn off location services on their mobile device. Notifications about GPS function are provided when the Plot Locator app is opened and in the Plot Locator User Guide, along with directions for modifying location services.

The GPS assist function is available to Plot Locator users on the “Enter Data” and “Locate Plot” screens (Figs. 1B, 2C). On the “Enter Data” screen, the green “GPS On” button indicates that the GPS function of the app is on (Fig. 1B). The GPS sensor will take a location reading and the coordinates will be saved when users click “Save.” On the “Locate Plot” screen, the “GPS Off” button indicates that GPS assist is off. This default state allows users to determine if and when they want the GPS plot and current coordinates and GPS accuracy (in meters) to be displayed alongside their plot notes and step and distance data. Users click the “GPS Off” button to activate the GPS assist. Once on, users can locate their plots by trying to match their current coordinates to their saved plot coordinates (Fig. 2C). The accuracy estimates and user's field notes should be considered when locating plots using GPS coordinates. The current GPS coordinates and accuracy estimate update every second within the app and when a new location is detected. GPS availability notifications are provided to users while GPS is functioning within the app. Because GPS frequently updates, the notifications are limited during use, but occur frequently enough to notify the user of important changes to GPS availability. The GPS update frequency is also affected by the default frequency rate of the user's mobile device.

For further location assistance, users can click the “Directions” button to use Google Maps (Fig. 2C). Once clicked, the desired plot coordinates are sent to Google Maps, and users can select the desired Google Maps directions options. The likely use case for Google Maps is to use the plot coordinates for study area and site location assistance (i.e., Google Maps will take you to the general area). Depending on plot size, and because of the current accuracy limitations of mobile devices, locating plots will still likely require the use of step and/or distance and field notes information.

### Compatibility

The Plot Locator app was developed for Android mobile devices, which are the most widely used mobile devices in the world (Savov, 2016). Plot Locator can be used with Android versions 4.1–8+. There are many different Android devices on the market with varying types of sensors and differences in how and where files are saved. We suggest that users read the Plot Locator User Guide and create a test file to test the app on their devices before using Plot Locator in the field. If the user's device saves to an SD card (or emulated SD card) using the /sdcard file path, then the app will save the test file to the SD card of the user's mobile device.

The CSV files created by the app can be imported into, and edited using, a wide variety of database and spreadsheet programs. Users can upload their CSV files into a variety of repositories such as the Environmental Data Initiative (EDI; https://environmentaldatainitiative.org/) and the Global Biodiversity Information Facility (GBIF; https://www.gbif.org/). Users may need to edit their CSV files to meet the specific data file criteria (e.g., format, save as a different file type) of the individual data repositories before uploading their data files.

### Field testing

The usability and accuracy of the Plot Locator app step counter and distance calculator were tested under different types of field conditions. A great deal of variation exists in individual field notes (e.g., the amount of detail required for a user to locate their plots), in mobile device GPS capabilities, and in field conditions that affect GPS function; therefore, we focused our tests on comparing the distance measured using a tape measure vs. pacing using the step and distance function of the app. The app was tested in both grassy fields and cypress swamps (*n* = 10 each) in Louisiana, USA. The tested distances were 10 m in length, simulating the number of steps from a landmark to a plot (i.e., distance data for a plot). The open grassy field sites were fairly level, whereas the cypress swamp sites had an uneven terrain and a forest canopy.

The Plot Locator app calculated distance with some variation in accuracy under different field conditions. The distance function successfully calculated distance under the grassy field conditions (app mean 9.9 m vs. tape mean 10 m; *t*‐test *P* > 0.05) (Table 2). The app slightly underestimated the actual distance traveled in the uneven terrain of the cypress swamp (app mean 9.1 m vs. tape mean 10 m; *t*‐test *P* < 0.0001). The accuracy difference between these two locations was likely due to the adjustments that the user made while walking in the different types of terrain (longer vs. shorter steps) and if the user rounded up the distance measurement. These results suggest that users should determine their step length under the field conditions in which they will be using the app and make alterations when the terrain conditions change. Users may also want to add condition notes for plots located in difficult terrain.

**Table 2:** Distances determined using the Plot Locator app vs. a tape measure in grassy field and cypress swamp sites.

Terrain	Site	Stride length (m)	Estimated app distance (m)	No. of app steps	Actual distance (m)	No. of steps	Difference (actual – estimate distance in m)
Field	1	0.66	9.9	15	10	15	0.1
Field	2	0.66	9.9	15	10	15	0.1
Field	3	0.66	9.9	15	10	15	0.1
Field	4	0.66	9.9	15	10	15	0.1
Field	5	0.66	9.9	15	10	15	0.1
Field	6	0.66	9.9	15	10	15	0.1
Field	7	0.66	9.9	15	10	15	0.1
Field	8	0.66	9.9	15	10	15	0.1
Field	9	0.66	9.9	15	10	15	0.1
Field	10	0.66	9.9	15	10	15	0.1
Cypress	1	0.7	9.1	13	10	13	0.9
Cypress	2	0.7	9.1	13	10	13	0.9
Cypress	3	0.7	9.1	13	10	13	0.9
Cypress	4	0.7	9.1	13	10	13	0.9
Cypress	5	0.7	9.1	13	10	13	0.9
Cypress	6	0.7	9.1	13	10	13	0.9
Cypress	7	0.7	9.1	13	10	13	0.9
Cypress	8	0.7	9.1	13	10	13	0.9
Cypress	9	0.7	9.1	13	10	13	0.9
Cypress	10	0.7	9.1	13	10	13	0.9

## CONCLUSIONS

Mobile apps such as Plot Locator can assist field biologists with a variety of tasks. The Plot Locator app leverages the power of mobile technology to help biologists create an organized searchable database of detailed plot location data that can be used to help them find their plots again in the field. The app provides a variety of functions that can assist field biologists with or without GPS access or online connectivity and may reduce the amount of equipment biologists need to take into the field. The notes function allows users to create detailed records of plot locations in relation to landmarks, including the distances between them calculated using a step count and stride length. The digital compass provides an additional location aid to enrich plot location notes and help biologists locate field plots. If biologists have access to GPS in the field, the app provides a GPS assist that records coordinates and helps users navigate to field plots. The recorded coordinates can also be sent to Google Maps, another assistive technology for locating previously sampled field sites and plots. We suggest that users test the app before using it in the field to be sure Plot Locator is compatible with their Android mobile device and to practice using the app to its fullest. If biologists wish to use mapping and tracking apps and have access to reliable cell tower, satellite, or Wi‐Fi signals in the field, then we suggest trying apps such as Fieldmap or Orux maps. The Plot Locator app, its source code, and a user guide are available for download from GitHub (https://github.com/jboudell/PlotLocator).

## Data Availability

The Plot Locator app, source code, and user guide are available for download from GitHub (https://github.com/jboudell/PlotLocator). The Plot Locator app is released under the GNU General Public License.
